# Targeting the SR-B1 Receptor as a Gateway for Cancer Therapy and Imaging

**DOI:** 10.3389/fphar.2016.00466

**Published:** 2016-12-15

**Authors:** Linda K. Mooberry, Nirupama A. Sabnis, Marlyn Panchoo, Bhavani Nagarajan, Andras G. Lacko

**Affiliations:** ^1^Institute for Cardiovascular and Metabolic Disease, University of North Texas Health Science Center, Fort WorthTX, USA; ^2^Department of Pediatrics, University of North Texas Health Science Center, Fort WorthTX, USA

**Keywords:** SR-B1, Drug Delivery Systems, tumor imaging, gateway for theranostics, individual therapy

## Abstract

Malignant tumors display remarkable heterogeneity to the extent that even at the same tissue site different types of cells with varying genetic background may be found. In contrast, a relatively consistent marker the scavenger receptor type B1 (SR-B1) has been found to be consistently overexpressed by most tumor cells. Scavenger Receptor Class B Type I (SR-BI) is a high density lipoprotein (HDL) receptor that facilitates the uptake of cholesterol esters from circulating lipoproteins. Additional findings suggest a critical role for SR-BI in cholesterol metabolism, signaling, motility, and proliferation of cancer cells and thus a potential major impact in carcinogenesis and metastasis. Recent findings indicate that the level of SR-BI expression correlate with aggressiveness and poor survival in breast and prostate cancer. Moreover, genomic data show that depending on the type of cancer, high or low SR-BI expression may promote poor survival. This review discusses the importance of SR-BI as a diagnostic as well as prognostic indicator of cancer to help elucidate the contributions of this protein to cancer development, progression, and survival. In addition, the SR-B1 receptor has been shown to serve as a potential gateway for the delivery of therapeutic agents when reconstituted high density lipoprotein nanoparticles are used for their transport to cancer cells and tumors. Opportunities for the development of new technologies, particularly in the areas of cancer therapy and tumor imaging are discussed.

## Background

**FIGURE 1:**
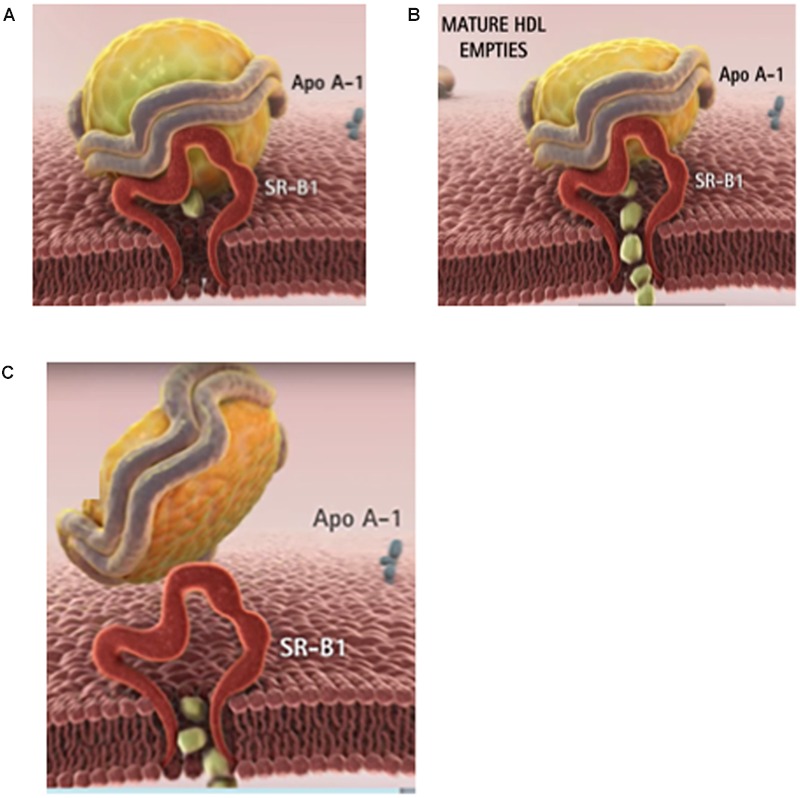
**The physiological role of HDL is to clear the excess tissue cholesterol through the liver. (A)** In phase 1, the cholesterol-rich HDL is captured by the SR-B1 receptor on the surface of the cell. **(B)** In phase 2, HDL delivers its cholesteryl ester cargo through SR-B1 directly into the cytoplasm. **(C)** In phase 3, the depleted HDL particle is released into the blood. The majority of cancer patients have lower than normal blood cholesterol especially HDL (or good) cholesterol. Images courtesy of Andras Lacko; full animation video is available at https://www.youtube.com/watch?v=q0YiPqmsXRg.

High density lipoproteins (HDLs) have been designated as “the good cholesterol” carriers due to their athero-protective effects. The beneficial impact of elevated HDL levels have been attributed to several factors, including participation in reverse cholesterol transport, anti-oxidant effects and anti-inflammatory functions (Miller et al., 1985; Navab et al., 1991, 2000a,b, 2001). During reverse cholesterol transport, HDL removes excess peripheral cholesterol and channels it toward the liver for disposal (or re-cycling), facilitated, at least in part, by the Scavenger Receptor, Class B, Type 1 (SR-B1; **Figure 1A**). Decreased plasma cholesterol levels (especially, lower HDL cholesterol levels) have been found in several groups of cancer patients representing a broad range of malignancies (Rose et al., 1974; Fiorenza et al., 2000; Shah et al., 2008; Muntoni et al., 2009). Regarding the mechanism of the lowering of HDL cholesterol levels in cancer patients, several reports found over-expression of the SR-B1 receptors as the potential cause (Lacko et al., 2002; Mooberry et al., 2010;

Shahzad et al., 2011; de Gonzalo-Calvo et al., 2015; Li et al., 2016; Yuan et al., 2016). Consequently, one of the key issues to be resolved is the extent to which the increased functionality of the SR-B1 receptor may be responsible for the markedly lower HDL cholesterol levels found in most cancer patients as the precise role of the SR-B1 receptor in the development of malignant and metastatic growth is yet to be delineated. Findings from a number of studies (Cruz et al., 2013; Yang et al., 2013) suggest that the delivery of cholesterol to rapidly growing malignant cells, facilitated by the overexpressed SR-B1 receptor, is essential for at least some tumor types. Cao et al. (2004) on the other hand showed that breast cancer xenografts could not develop when the carboxy terminal of the CLA-1 (SR-B1) receptor was mutated while other studies indicated that the carboxy terminal region of the SR-B1 receptor was not directly involved in cholesterol transport (Connelly et al., 2001; Cao et al., 2004). While the mechanism whereby the SR-B1 receptor contributes to carcinogenesis is not yet clear, expression of the SR-B1 receptor may signal the prognosis and survival prospects of cancer patients and thus could have a role as an important diagnostic biomarker for specific malignancies.

## HDL Metabolism and Receptors with Special Emphasis on Scavenger Receptors

The precursor to circulating HDL is produced in the liver and subsequently released into the circulation. These are cholesterol-poor, discoidal structures, composed primarily of apolipoprotein A-I (apo A-I) and phospholipid (Hamilton et al., 1976). Next the ATP-binding cassette protein, ABCA-1, facilitates the loading of the nascent discoidal HDL with excess cholesterol from peripheral tissues. The cholesterol progressively accumulated in HDL is then esterified by Lecithin-Cholesterol Acyltransferase (LCAT) to produce mature spherical HDL particles with an expanded core region, containing primarily cholesteryl esters. Although apo A-I is the main protein component of HDL, it acquires other apolipoproteins including apo A-II, apo A-IV, apo C-III or apo E, from other lipoproteins. The respective apolipoproteins may facilitate the interactions of HDL with several lipoprotein receptors (Alaupovic et al., 1972; Scanu, 1972; Scanu and Wisdom, 1972; Schmitz and Mollers, 1994; Fidge, 1999).

Unlike the delivery of Low density lipoprotein (LDL) cholesterol that involves endocytic uptake of the entire lipoprotein particle, most of the cholesterol from HDL undergoes a process termed selective lipid uptake, facilitated by the SR-B1 receptor, involving only the removal of cholesteryl esters from the HDL complex (**Figure 1B**). The subsequent steps involve the release of the now cholesterol-depleted HDL via a process called retroendocytosis (Pagler et al., 2006; Sun et al., 2006). The protein component of HDL is thus not internalized, but remains (at least transiently) bound to the plasma membrane (**Figure 1C**).

A plasma membrane protein, SR-B1, is the murine HDL receptor (Acton et al., 1996). The human homolog is termed CD36-and-LIMPII analogous-1 (CLA-1). The human HDL receptor is composed of 509 amino acids and has an apparent molecular weight of 85 kDa including carbohydrate moieties that are attached to the polypeptide portion via N-linked glycosylation (Calvo et al., 1997). Expression of SR-B1 is most prominent in tissues involved in cholesterol metabolism or steroid hormone synthesis, including the liver, adrenal glands, ovaries, and testes (Landschulz et al., 1996; Calvo et al., 1997; Arenas et al., 2004; Shahzad et al., 2011). However, besides acting as an HDL receptor, facilitating selective lipid uptake from HDL, SR-B1 has also been shown to be a high-affinity receptor for LDL, VLDL, oxidized LDL, and acetylated LDL (Calvo et al., 1997).

The mechanism of selective lipid uptake by SR-B1 has yet to be described in detail. The known domains of SR-B1 consist of a short N-terminal intracellular region, an N-terminal transmembrane domain, a cysteine-rich region that forms an extracellular loop, a C-terminal transmembrane domain, and a C-terminal intracellular domain (Krieger, 1999). It has been postulated that SR-B1 forms a “hydrophobic channel” to allow passage of lipophilic HDL components into the cell (Rodrigueza et al., 1999). Reports in the literature have attempted to delineate the respective functional domains and their specific contributions to HDL binding, and selective lipid uptake. Studies creating chimeric receptors between domains of CD36 and SR-B1 have shown that neither the N-terminal nor C-terminal transmembrane and cytoplasmic domains of SR-B1 are necessary for selective lipid uptake. The function of selective lipid uptake was found to be the task of the extracellular domain of SR-B1 (Connelly et al., 2001). The extracellular domain of SR-B1 contains six cysteine residues highly conserved among mouse, rat, hamster, rabbit, pig, cow, dog, and human isoforms (Hu et al., 2011). In a mutational analysis, some of these cysteine residues were found to interfere with cell surface localization and selective lipid uptake, possibly due to the formation of aberrant disulfide bonds and protein misfolding (Hu et al., 2011).

## Expression and Potential Roles of the SR-B1 Receptor in Cancer Cells and Tumors

### Cholesterol Metabolism

A breast cancer cell line HBL-100, was shown to take up cholesteryl esters (CE) selectively through SR-B1. The cells internalized CE in a dose-dependent manner in response to the HDL_3_ concentration. No increase in uptake of CE was observed with adrenocorticotropic hormone, but down-regulation of SR-B1 expression and decreased CE uptake were seen with phorbol 12-myristate 13-acetate (PMA). The cholesteryl esters were subsequently hydrolyzed by hormone sensitive lipase (Pussinen et al., 2000). A study in patients with three subtypes of breast cancer found that CE accumulation corresponded with increases in cholesterol esterification and the expression of the LDL receptor and SR-B1 (de Gonzalo-Calvo et al., 2015). In patients with Her-2 breast cancer (ER-/PR-/HER2+) or triple negative breast cancer (ER-/PR-/HER2-), tumor samples with enriched cholesteryl ester accumulation were associated with increased expression of the cell proliferation marker, Ki-67, thus indicating the potential for enhanced tumor proliferation and progression. However, the third subtype, estrogen and progesterone receptor positive luminal-A breast cancer (ER+/PR+/HER2-) did not show increased intracellular cholesteryl esters or increased Ki-67 expression (de Gonzalo-Calvo et al., 2015). Because of the differential cholesteryl ester accumulation and expression of SR-B1 in varying grades and subtypes of breast cancer that links to proliferation and progression, more research into cholesterol metabolism in cancer subtypes is warranted.

In a study involving high grade metastatic prostate cancer increased accumulation of CE into lipid droplets was observed. The CE accumulation was found to be in response to the loss of the tumor suppressor PTEN, activation of the mTOR pathway and downstream activation of SREBP, and the up-regulation of LDL receptor (Yue et al., 2014). This study examined only uptake of LDL and the LDL receptor, not selective CE uptake through SR-B1. However, the SR-B1 gene does contain two Sterol Regulatory Elements (SREs) that are activated by SREBP (Lopez and McLean, 1999). In a report on high Gleason grade primary prostate cancer, SR-B1 mRNA and protein expression were found to be high, unlike the LDL receptor (Schörghofer et al., 2015). The link between SR-B1 expression and cholesteryl ester accumulation could provide the link between androgen independence and advanced prostate cancer (Twiddy et al., 2012; Schörghofer et al., 2015).

### Signaling Mechanisms

High density lipoprotein, beyond binding to SR-B1 and completing reverse cholesterol transport has also been found to subsequently activate the PI3K/Akt and MAPK pathways (Danilo et al., 2013). SR-B1 has been investigated as a mediator of cell signaling events in atherosclerosis (Al-Jarallah and Trigatti, 2010; Saddar et al., 2010). SR-B1 has been found to be highly expressed in several different types of tumors including breast, ovarian, colorectal, pancreatic cancer (Shahzad et al., 2011) and prostate cancer (Schörghofer et al., 2015) with relatively low expression in most healthy tissue (Rhainds and Brissette, 2004). Although SRB1 has been found to have a protective role in atherosclerosis, high expression of SR-B1 has also been linked to the rapid progression and aggressiveness of certain types of cancer, while the exact underlying mechanisms and downstream cell signaling events of SR-B1 are presently not fully understood.

Hypercholesterolemia and excessive consumption of a cholesterol or fat rich diet have been recognized as risk factors for prostate cancer. Additional studies have also shown that the aggressive form of the disease is likely to be associated with the intracellular accumulation of esterified cholesterol (Shimizu et al., 1991; Bravi et al., 2006; Yue et al., 2014). While cellular cholesterol uptake is known to be facilitated by the SR-B1 and the LDL receptor (Acton et al., 1996; Krieger, 1999; Chen and Hughes-Fulford, 2001; Rohrl and Stangl, 2013). SR-B1 has been found to be highly expressed in prostate cancer cells (Connelly and Williams, 2004; Kraemer, 2007; Connelly, 2009; Hoekstra et al., 2009). Twiddy et al. (2012) have found that protein expression of SR-B1 was significantly increased upon progression to castration-resistance stage in the LNCaP xenograft model (Leon et al., 2010). Even higher expression of SR-B1 has been reported in C4-2 cells compared to LNCaP cells, suggesting a role for SR-B1 in the development and progression of advanced prostate cancer (Twiddy et al., 2012). These findings indicate that the castration resistant prostate cancer cells might be able to survive in a low androgen environment by increasing their demand for cholesterol influx for cellular processes (Wu et al., 1994; Leon et al., 2010).

The Akt signaling pathway is activated in 40% of breast cancers (Bellacosa et al., 2005). Studies conducted by Danilo et al. (2013) showed that HDL (100 μg/ml) can trigger the activation of Erk1/2 and Akt pathways in breast cancer cells. Their studies in MDA-MB-231 cells have shown that knockdown of the SR-B1 receptor (via lentiviral transduction of SR-B1 shRNA or pharmacological inhibition of SR-B1 in the presence of HDL) mitigated the activation of the expression of the Akt signaling molecule. Whereas, the knockdown of SR-B1 receptor in MCF-7 cells showed a marked decrease in the activation of Akt and the Erk1/2 pathway. These findings support the hypothesis that the binding of HDL to SR-B1 may be involved in the regulation of these signaling pathways. Additionally, the studies of Danilo et al. (2013) have also shown that knockdown of SR-B1 in MDA-MB-231 cells triggered a reduction in the intracellular cholesterol levels, in concurrence with the studies of Assanasen et al. (2005) showing that SR-B1 modulated signal transduction could be linked to cellular cholesterol flux (Danilo et al., 2013).

### Proliferation

Cancer cells are considered to be dependent on cholesterol for their proliferation and rapid progression (Gospodarowicz et al., 1982; Johnson et al., 1991; Cruz et al., 2013). Comparative analysis of SR-B1 expression in breast tissues revealed a high expression of SR-B1 in breast tumors compared to the healthy surrounding tissue (Cao et al., 2004; Shahzad et al., 2011). Additionally, reports from multiple studies, conducted with several cancer cells revealed that HDL can enhance their capacity for proliferation and migration (Gospodarowicz et al., 1982; Jozan et al., 1985; Rotheneder and Kostner, 1989; Pussinen et al., 2000; Pan et al., 2012a,b). Studies conducted by Danilo et al. (2013) in MDA-MB-231 (triple negative breast cancer cells) have shown that pharmacological inhibition of SR-B1 (through BLT-1) or knockdown of SR-B1 resulted in a reduction in cell proliferation perhaps via a decreased activation of the Akt signaling pathway.

### Migration

High density lipoprotein, a carrier of plasma cholesterol has also been found to be a signaling molecule inducing the migration of endothelial cells by activating the Akt and MAPK pathways (Danilo et al., 2013). Similarly, Danilo et al. (2013) have shown that HDL can induce the migration of MCF-7 and MDA-MB-231 cells. In addition, Danilo et al. (2013) performed SR-B1 knockdown in MDA-MB-231 cells and found a significant decrease in their potential for migration (1.65-fold) compared to control cells. These findings are in accordance with studies conducted in nasopharyngeal carcinoma cells, where knockdown of SR-B1 resulted in reduced migration in 5-8F shSR-B1-treated cells compared to control cells (Zheng et al., 2013).

### Angiogenesis

**FIGURE 2:**
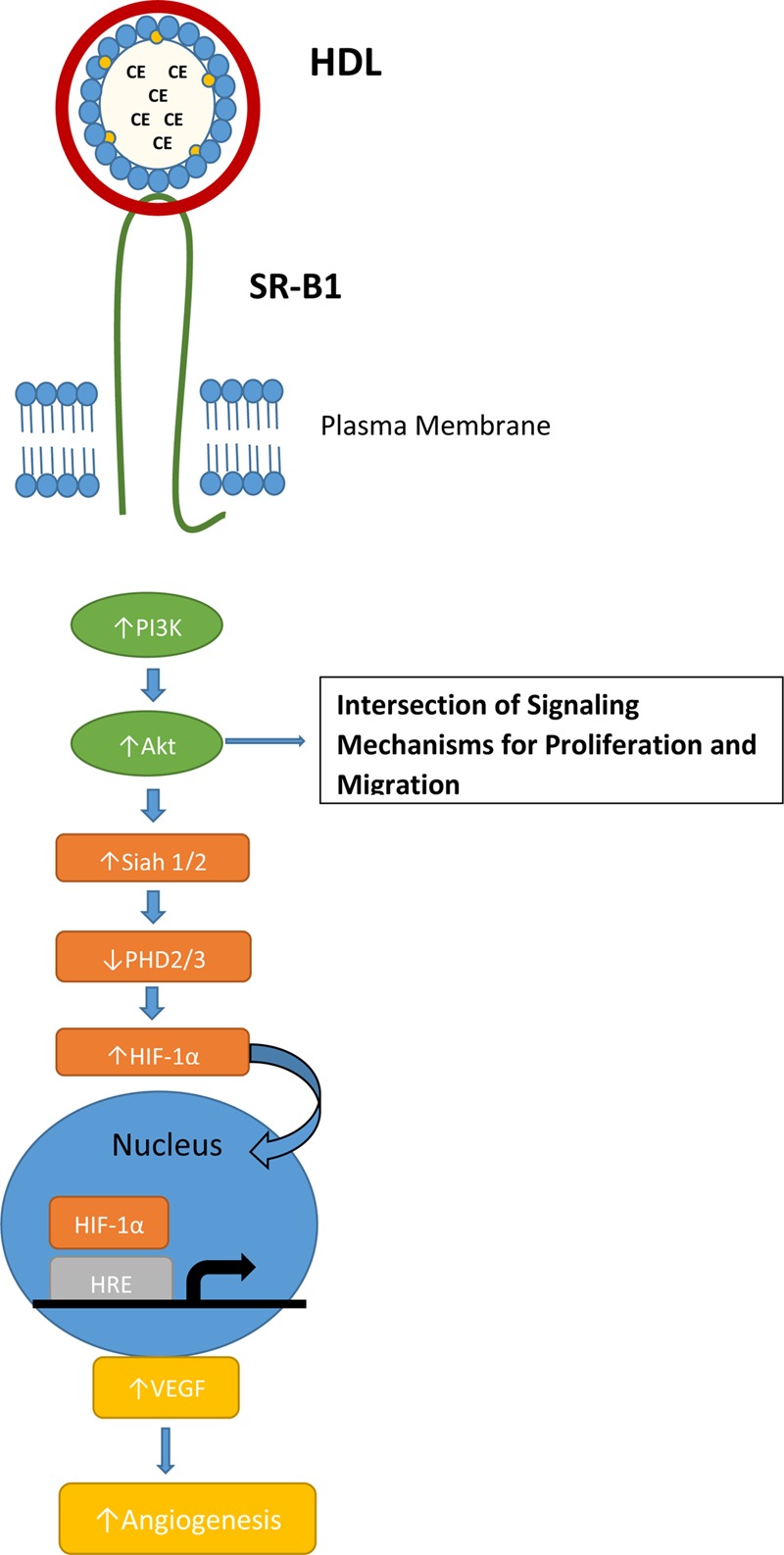
**SR-B1 signaling pathway for normal hypoxia-induced angiogenesis.** Interaction of HDL and SR-B1 activates the PI3K/Akt pathway leading to HIF-1α translocation to the nucleus and transcription of VEGF. HDL treatment and over-expression of SR-B1 has been shown to activate the PI3K/Akt pathway in tumor cells and stimulate proliferation and migration.

High density lipoprotein was shown to induce endothelial tube formation and angiogenesis in human coronary artery endothelial cells through a MAPK pathway (Miura et al., 2003). Seetharam et al. (2006) showed that endothelial cell migration was activated by PI3K/Akt/MAPK pathways mediated by SR-B1. This effect was impaired in SR-B1 knockout mice (Seetharam et al., 2006). In hypoxic conditions, HDL stimulated the PI3K/Akt pathway through SR-B1, resulting in stabilization of hypoxic inducible factor-1α (HIF-1α; **Figure 2**). The result is activation of transcription of VEGF and induction of angiogenesis, which could be inhibited by transduction with a lentivirus containing a shRNA against SR-B1 (Tan et al., 2014). Mutational analysis of SR-B1 found that changing a critical glutamine residue in one of the transmembrane domains disrupted p38/MAPK signaling. In a mouse model system, the mutant SR-B1 was not capable of increasing endothelial cell abundance or stimulating angiogenesis, even with HDL activation (Saddar et al., 2013). In inflammatory-mediated angiogenesis, HDL and SR-B1 appear to play an inhibitory role (McGrath et al., 2009).

Like HDL and SR-BI, the signaling mechanism for angiogenesis is often utilized by tumors via PI3K/Akt activation and the HIF-1α pathway (Karar and Maity, 2011). Several studies have identified a link between SR-BI and the PI3K/Akt pathway, but the mechanism where by SR-BI impacts angiogenesis is not clear. Danilo et al. (2013) found that mice bearing a MDA-MB-231 xenografts had significantly increased microvessel density in the control tumor when compared to the SR-B1 knockdown tumor. In the TRAMP mouse model of prostate cancer, mice fed a high-fat, high-cholesterol Western-style diet had decreased plasma HDL cholesterol levels, increased tumor expression of SR-B1 and increased tumor angiogenesis (Llaverias et al., 2010).

These studies in cholesterol metabolism, signaling mech-anism, proliferation, migration, and angiogenesis have been conducted with cancer cells, tumor xenograft in mouse models, and patient tumor samples of prostate and breast cancer. The majority of the findings reported in the literature regarding SR-B1 and cancer focus on breast and prostate cancer. Expanding the focus of these studies to other types of tumors would allow the elucidation of the role of the SR-B1 receptor and the related signaling mechanisms governing cancer proliferation, migration, invasion, and angiogenesis.

## SR-B1 as a Predictor of Tumor Prognosis and Survival

Scavenger Receptor Class B Type 1 (SR-B1) is a HDL receptor that facilitates the uptake of cholesterol esters from circulating lipoproteins (primarily from HDL). Recent findings indicate that the level of SR-B1 expression is correlated with aggressiveness and poor survival in breast and prostate cancer (Schörghofer et al., 2015; Yuan et al., 2016). Moreover, genomic data show that depending on the type of cancer, high or low SR-B1 expression may promote poor survival. This section focuses on SR-B1 as a diagnostic as well as prognostic biomarker for specific types of cancer to facilitate better outcome for cancer patients by allowing the tailoring of individual therapy via the assessment of progression and survival.

An elevated expression of SR-B1 has been observed in a wide variety of malignant cell lines such as breast, prostate, ovarian, pancreatic, nasopharyngeal, and colorectal cancers (Martin et al., 1999; Cao et al., 2004; Shahzad et al., 2011; Twiddy et al., 2012; Zheng et al., 2013). In human breast carcinoma, higher SR-B1 protein levels were observed in malignant tissue versus cancer free surrounding tissue in xenograft studies (Cao et al., 2004). Moreover, mutation of the HDL receptor attenuated the proliferation of MCF-7 cells (Cao et al., 2004). Similarly, another study demonstrated that over 50% of all examined breast cancer tissue showed a high expression of SR-B1 which significantly correlated with larger size of the tumor, metastasis to lymph node, and overall reduced survival (Yuan et al., 2016). Additionally, breast carcinomas displaying higher SR-B1 mRNA and protein levels showed enhanced accumulation of intratumor cholesteryl ester which linked to aggressiveness and poor prognosis in patients (de Gonzalo-Calvo et al., 2015). Likewise, knockdown of SR-B1 significantly reduced tumor size in mouse models and reduced cell proliferation and migration *in vitro*. This study also identified a link between HDL-SR-B1 and Akt where breast cancer cells stimulated with cholesteryl ester from HDL exhibited continued proliferation, migration/invasion and subsequent tumor growth (Danilo et al., 2013). Taken together these studies reveal a critical role for SR-B1 as a prognostic marker and therapeutic target for breast cancer treatment.

The uptake of cholesterol ester from HDL via SR-B1 facilitates many important cellular functions such as the production of androgens. Thus, a knockdown of SR-B1 was shown to diminish prostate-specific antigen (PSA) levels and cell viability in prostate cancer cells (Twiddy et al., 2012). Using mRNA microarray files and immunohistochemical analysis of prostate cancer tissue sections Schörghofer et al. (2015) found that mRNA and protein SR-B1 expression positively correlated with progression and metastasis of prostate cancer. The study revealed a higher SR-B1 expression in higher Gleason grade prostate tumors than in lower Gleason grade prostate tumors (Schörghofer et al., 2015). Additionally, SR-B1 expression and enzymes involved in the synthesis of androgens exhibited a positive correlation indicating that SR-B1 may have a potential role in establishing androgen independence (Schörghofer et al., 2015). Moreover, in nasopharyngeal cancer (NPC) SR-B1 was overexpressed in 75% of clinical NPC samples and all examined NPC cell lines while SR-B1 expression was lower in surrounding non-malignant epithelial cells suggesting SR-B1 as a possible biomarker of NPC (Zheng et al., 2013).

Interestingly the generation of Kaplan–Meier survival curves using the R2 Genomics Analysis and Visualization Platform^1^ along with publicly accessible microarray data confirmed the correlation of high SR-B1 expression and poor prognosis in neuroblastoma, Ewing sarcoma, bladder, breast, and myeloma cancers (Pawitan et al., 2005; Hanamura et al., 2006; Molenaar et al., 2012; Sjodahl et al., 2012). However, a reverse was observed in cancers such ovarian, osteosarcoma, B-Cell lymphoma, and lung (Bild et al., 2006; Kuijjer et al., 2012; Lisowska et al., 2014), specifically low SR-B1 correlated with poor survival. Given the complexities involved in the regulation of SR-B1 expression it is difficult to hypothesize regarding mechanism(s) involved in the observed variations of SR-B1 expression in relation to patient survival. Perhaps transcription factors such as p53, STAT proteins and c-myc could play a role in the regulation of SR-B1 expression (Gutierrez-Pajares et al., 2016) thus impacting carcinogenesis and metastasis. Alternatively, microRNAs such as miR-125 and miR455 may decrease the expression of SR-B1 mRNA and protein levels in malignant tumors as they do in steroidogenic tissue (Hu et al., 2012). Consequently, all these factors (alone or together) could account for the observed variations in SR-B1 expression and its correlation with overall patient survival.

## SR-B1 as a Gateway for Drug Delivery

The first definitive role for HDL in reverse cholesterol transport as a promoter of the excess cholesterol from peripheral cells to the liver was proposed by Glomset (1968). In this process, the cholesterol from the circulating HDL particles can be taken up via receptor-mediated mechanisms, either directly from HDL through SR-B1 or by the LDL receptor, subsequent to the transfer of cholesteryl esters from HDL (Besler et al., 2012). The major difference in the uptake of HDL payloads via LDL and SR-B1 receptors is that the LDL mediated operation is endocytotic (non-specific) whereas SR-B1 mediated uptake is non-endocytotic (highly specific). In the later process, the lipoprotein specifically binds to the cell surface and delivers the hydrophobic cargo directly inside the cells, without internalizing the lipoprotein shell. This mechanism bypasses the reticulo endothelial system and lysosomal exposure of the payload (including drugs) and is, therefore, preferred compared with the endocytotic process utilized by the LDL receptors for drug delivery (Bricarello et al., 2011; Ng et al., 2011; Cruz et al., 2013; McMahon et al., 2015).

Due to their unique structure of a phospholipid monolayer stabilized by a protein component, covering a firm inner hydrophobic core, lipoproteins and especially HDL, are considered as natural nanocarriers to harbor and transport hydrophobic compounds, including anti-cancer drugs to cancer cells and tumors via the SR-B1 gateway. (Counsell and Pohland, 1982; Bijsterbosch and van Berkel, 1990; Bijsterbosch et al., 1994; Firestone, 1994; Lacko et al., 2007; Bricarello et al., 2011). Silvente-Poirot and Poirot (2012) have demonstrated that carcinogenesis and tumor development have a dramatic impact on alterations in cholesterol metabolism of the cells. As a repercussion, there is an enhanced expression of SR-B1 receptors in most malignant cells (Shahzad et al., 2011) to meet their cholesterol needs for enhanced proliferative rates (Mooberry et al., 2010; Ng et al., 2011; Shahzad et al., 2011; Zheng et al., 2013). Thus the SR-B1 receptor has a great potential to be a biomarker of different types of cancers. Several studies have shown that malignant cells have an elevated requirement for cholesterol resulting in reduced HDL cholesterol levels in cancer patients, as compared with healthy subjects (Lacko et al., 2007; Ng et al., 2011). Using a quantitative real-time PCR technique, Shahzad et al. (2011) have demonstrated considerable increase in the expression of SR-B1 receptors in most tumor samples of breast, ovarian, colorectal and pancreatic cancer compared with those of normal tissues (Shahzad et al., 2011). The SR-B1 mediated process has also been shown to function in the uptake of anticancer agents from reconstituted high density lipoprotein (rHDL) by cancer cells (McConathy et al., 2008; Mooberry et al., 2010; Sabnis et al., 2012; Shen et al., 2014; Song et al., 2015). An enhanced uptake of paclitaxel from rHDL nanoparticles was demonstrated in SR-B1 overexpressing prostate cancer cells by Mooberry et al. (2010) where the SR-B1 mediated entry of the drug represented 82% of the total paclitaxel incorporation by these cells. In another study, using a construct of a synthetic HDL-gold nanoparticle template, Yang et al. (2013) showed that similar to natural HDLs, biomimetic HDL-NPs can be formulated to target SR-B1 receptors in lymphoma cells. Furthermore, by differential manipulation of SR-B1 binding and restricting cholesterol delivery they showed induction of apoptosis in lymphoma cells (Yang et al., 2013).

## SR-B1 as a Conduit for the Delivery of Imaging Agents

Due to their capacity for incorporating preferentially hydrophobic compounds in their core region, lipoproteins have been well-recognized as platform for delivering theranostic agents. rHDL-like particles have been developed for imaging of atherosclerosis by contrast enhanced MRI (Frias et al., 2004). In this study, ApoA-I, extracted from human plasma, was reconstituted with commercially available phospholipids, which was coupled with a gadolinium (Gd)-chelate, making the particle visible for MRI. Furthermore, a green-emitting and amphiphilic fluorophore, 7-nitro-2-1,3-benzoxadiazol-4-yl (NBD-DPPE) was included in the lipid monolayer, to enable its detection with fluorescence techniques. MRI scans of the abdominal aorta of the apo E knock out mice injected with this preparation revealed significant accumulation of rHDL in the aortic wall. Extending this approach, Cormode et al. (2008) developed a synthetic HDL/apoA-I mimicking nanoparticle composed of lipids and an apo A-I derived, amphiphatic α-helical peptide, 37pA. These nanoparticles were formulated with Gd-chelates and a lipid-based fluorophore, rhodamine-PE, into the phospholipid layer, making the particles suitable for MRI and fluorescence imaging.

Skajaa et al. (2010) employed rHDL as a vector for delivering a variety of diagnostic agents to susceptible atherosclerotic plaques in a mouse model. They showed that by loading various types of image-enhancing compounds into either the core or surface of rHDL nanoparticles, they can be visualized by different imaging modalities (MRI, CT, optical). These findings suggest that imaging of pathological processes other than atherosclerosis may also be accomplished using rHDL as a carrier.

Fluorescence based studies reported by Shah et al. (2016) showed that the drug valrubicin has differential optical properties when encapsulated in rHDL. Moreover, valrubicin has low quantum yield in aqueous environment and it increases several fold when incorporated into rHDL nanoparticles. Further, they showed that the rHDL-valrubicin formulation could be used for confocal imaging in a SR-B1-positive prostate cancer cell line.

These studies reveal the potential of HDL type nanoparticles to deliver a cargo of theranostic agents via SR-B1 receptors; thus further emphasizing the role of the SR-B1 receptors in facilitating the delivery of imaging agents.

## Cancer Therapeutics: Challenges and Novel Therapies

Many of the currently employed cancer chemotherapeutic drugs are associated with challenges, including solubility, bio-distribution and tissue non-specificity (Farokhzad and Langer, 2009). The majority of anti-cancer drugs are also non-selective in their bio-distribution as they affect all rapidly dividing cells thus disseminating the drug into both healthy as well as malignant tissues. Advances in nanotechnology and using targeted drug delivery vehicles, have shown great potential to overcome these challenges to effective chemotherapy. The therapeutic index of a drug can be significantly improved by increasing the preferential accumulation of the therapeutic agent in malignant cells while protecting healthy cells and thereby reducing toxic side effects (Rigotti et al., 2003; Foit et al., 2015; Lacko et al., 2015).

High density lipoprotein HDL-type nanocarriers exhibit many features of an ideal drug-delivery vehicle. The particles have small size, are biocompatible, non-immunogenic, have long circulating half-life and exhibit selective delivery (Zheng et al., 2005). Thus, HDL particle functionality is a primary tool for HDL-based drug delivery strategies (Kypreos et al., 2013). The potential of HDL-type nanoparticles for drug delivery was proposed by Counsell and Pohland (1982); however, systematic studies on this were first carried out by Kader and Pater (2002) after 20 years (Counsell and Pohland, 1982). They have reported that loading anticancer drugs into HDL as well as LDL had minimal effect on the properties of the complexes, while the encapsulated drugs showed enhanced cytotoxicity toward human carcinoma cells (Kader and Pater, 2002). It has been well documented that tumor tissues are characterized by a leaky vasculature and low lymphatic drainage, leading to unequal interstitial pressure between different parts of the tumor (Wu et al., 2013; Omidi and Barar, 2014). This pressure difference leads to uneven retention of particles between the center of the tumor and its periphery. HDL and HDL-like particles are believed to exhibit active targeting via SR-B1 receptors instead of utilizing passive targeting through the enhanced permeability and retention (EPR) effect (Bricarello et al., 2011; Llaverias et al., 2011; Gorin et al., 2012; Danilo et al., 2013; Huynh and Zheng, 2013).

Although numerous antineoplastic drugs have shown promise during pre-clinical studies, the success rate of these drugs clearing clinical trials is extremely low. This is primarily due to the off target effects that many of these agents exhibit against peripheral (normal) tissues^2^. The selective delivery of hydrophobic drugs, specifically to malignant tumors is facilitated by SR-B1 receptor, and thus makes the rHDL drug-delivery system unique and a potential enhancer of cancer therapeutics. Lou et al demonstrated a preferential cytotoxicity of the antineoplastic drug aclacinomycin encased in rHDL toward malignant cells (Lou et al., 2005). Sabnis et al. (2012) found that valrubicin when encapsulated into rHDL nanoparticles was effective 1.8 and 2.6 times lower minimum inhibitory concentrations (IC50) than the free drug against prostate and ovarian cells, respectively. The valrubicin/rHDL formulation was also found to have reduced toxicity toward non-malignant cells, demonstrating the selective tumor delivery capabilities of rHDL nanoparticles. In the past 10 years, scientists are realizing the potential of HDL and HDL mimetic drug delivery strategies in cancer theranostics, primarily due to the interaction of these nanoparticles (**Figure 1**) with the SR-B1 receptor (McMahon et al., 2011; Jia et al., 2012; Rui et al., 2013; Zheng et al., 2013; Dong et al., 2014; Fischer et al., 2014).

## Future Opportunities for the Development of Cancer Biomarkers, Therapy and Imaging

Traditional chemotherapy has always been aﬄicted with long term and short term unpleasant side effects. In some cases the side effects are worse than the disease itself. It is well documented by different research groups now that malignant cells consistently overexpress SR-B1 receptors as compared to normal cells. Lipoprotein-based technologies could address the above concerns via their ability to selectively deliver their payload to cancer cells and tumors. Safety of apoA-1 containing HDL-type formulations for human systemic administration has been confirmed by numerous clinical studies (Lacko et al., 2002; Kuai et al., 2016). Additionally, these formulations have a potential to reposition several drugs that have been considered as anticancer drugs but have been disapproved due to poor solubility and excessive peripheral toxicity. This strategy could be commercially beneficial, in contrast with conventional drug- and target-screening strategies.

If determination of SR-B1 expression levels is included as a pretest before starting the chemotherapy regimen, the HDL based drug delivery could be tailored to respective cancer patients with high SR-B1 expressing tumors. Alternatively for low SR-B1 expressing patients, specific functionalized rHDL nanoparticles could be designed that will target different overexpressing tumor antigens. Thus the oncogenic drugs could be rerouted from the natural lipoproteins receptors thereby personalizing HDL-based therapeutics.

Although the concept of using lipoproteins as targeted drug-delivery agents was introduced back in 1982, and the suitability of their functional characteristics for pharmaceutical formulations was well known, there are currently no lipoprotein-based drug delivery formulations in clinical trials or in clinical use. However, considering their versatility and selective-targeting capability, biofunctional HDL-type nanoparticles are likely to bring a shift in the paradigm of next generation cancer theranostics. The SR-B1 receptor-mediated gateway of lipophilic anticancer drugs or imaging agents via HDL and HDL-mimetic nanoparticles has strengthened the potential for these preparations as theranostic agents.

## Author Contributions

All authors listed, have made substantial, direct and intellectual contribution to the work, and approved it for publication.

## Conflict of Interest Statement

AL is a co-inventor on two issued patents that involve SR-B1 mediated drug delivery. NS is a co-inventor on one issued patent that involves SR-B1 related drug delivery. All the other authors declare that the research was conducted in the absence of any commercial or financial relationships that could be construed as a potential conflict of interest.

## Footnotes

1 http://r2.amc.nl

2 https://www.bio.org/media/press-release/new-study-shows-rate-drug-approvals-lower-previously-reported-0
